# Muscle oxygenation as an indicator of shock severity in patients with suspected severe sepsis or septic shock

**DOI:** 10.1371/journal.pone.0182351

**Published:** 2017-08-03

**Authors:** Kenneth A. Schenkman, David J. Carlbom, Eileen M. Bulger, Wayne A. Ciesielski, Dana M. Fisk, Kellie L. Sheehan, Karin M. Asplund, Jeremy M. Shaver, Lorilee S. L. Arakaki

**Affiliations:** 1 Division of Critical Care Medicine, Department of Pediatrics, University of Washington, Seattle, Washington, United States of America; 2 Department of Bioengineering, University of Washington, Seattle, Washington, United States of America; 3 Department of Anesthesiology, University of Washington, Seattle, Washington, United States of America; 4 Division of Pulmonary and Critical Care, Department of Medicine, University of Washington, Seattle, Washington, United States of America; 5 Department of Surgery, University of Washington, Seattle, Washington, United States of America; 6 Eigenvector Research, Inc., Wenatchee, Washington, United States of America; Hospital Sirio-Libanes, BRAZIL

## Abstract

**Purpose:**

The aim of this pilot study was to evaluate the potential of a new noninvasive optical measurement of muscle oxygenation (MOx) to identify shock severity in patients with suspected sepsis.

**Methods:**

We enrolled 51 adult patients in the emergency department (ED) who presented with possible sepsis using traditional Systematic Inflammatory Response Syndrome criteria or who triggered a “Code Sepsis.” Noninvasive MOx measurements were made from the first dorsal interosseous muscles of the hand once potential sepsis/septic shock was identified, as soon as possible after admission to the ED. Shock severity was defined by concurrent systolic blood pressure, heart rate, and serum lactate levels. MOx was also measured in a control group of 17 healthy adults.

**Results:**

Mean (± SD) MOx in the healthy control group was 91.0 ± 5.5% (*n* = 17). Patients with mild, moderate, and severe shock had mean MOx values of 79.4 ± 21.2%, 48.6 ± 28.6%, and 42.2 ± 4.7%, respectively. Mean MOx for the mild and moderate shock severity categories were statistically different from healthy controls and from each other based on two-sample *t*-tests (p < 0.05).

**Conclusions:**

We demonstrate that noninvasive measurement of MOx was associated with clinical assessment of shock severity in suspected severe sepsis or septic shock. The ability of MOx to detect even mild septic shock has meaningful implications for emergency care, where decisions about triage and therapy must be made quickly and accurately. Future longitudinal studies may validate these findings and the value of MOx in monitoring patient status as treatment is administered.

## Introduction

Early identification of severe sepsis is one of the most important factors in improving clinical outcome [1]. In 2001, the introduction of early goal-directed therapy (EGDT) [2] began a systematic approach to treatment of the patient with suspected severe sepsis. The Society of Critical Care Medicine launched the Surviving Sepsis Campaign in 2004, and has since refined guidelines for this care [1]. However, despite effort focused on these issues, a good clinical indicator that can accurately identify inadequate perfusion in severe sepsis and that can serve as an index from which to titrate therapy remains elusive. The lack of an accurate marker for hypoperfusion in sepsis was one factor leading recent multicenter studies to question the efficacy of the original EGDT protocol [3, 4]. The need for a new clinical marker identifying hypoperfusion in patients with severe sepsis or septic shock fueled the design of this study.

Optimal sepsis management in the emergency department (ED) and intensive care unit (ICU) requires knowledge of oxygen delivery and extraction. Currently, central venous oxygenation (ScvO2) is a component of EGDT that measures systemic oxygenation. ScvO2 monitoring remains controversial [3] and it is technically challenging to implement [5]. Relatively infrequent use of ScvO2 monitoring has been identified in patients at risk for septic shock in EDs [5], and when used, uncertainty in interpretation remains [6]. Lactate is currently considered the best marker for sepsis severity and is recommended in patients with suspected sepsis [7–10]. Unlike ScvO2, lactate levels lag behind changes in clinical condition since the accumulation or clearance of lactate are metabolic responses to clinical states.

We have developed a noninvasive device based on optical spectroscopy that has the potential to provide accurate and continuous measurements of muscle oxygenation (MOx). Our device measures full optical spectra in the visible and near infrared (NIR) wavelength regions, in contrast to near infrared spectroscopy (NIRS) devices that measure 2–6 selected wavelengths in the NIR. Measurement and analysis of full spectra yields MOx measurements that are accurate [11] and potentially sensitive to the perfusion state of muscle. The indication of the presence and the degree of tissue hypoxia by MOx may be clinically useful since microcirculatory derangement in sepsis likely leads to progressive hypoperfusion and development of organ dysfunction [12–15]. The goal of this pilot study was to understand the relationship between MOx and traditional measures of shock severity in patients with suspected severe sepsis or septic shock.

## Methods

### Study design, setting and population

This was a prospective, observational pilot study conducted in a major academic regional referral and Level 1 trauma center. The protocol was approved by the institutional review board of the University of Washington. In a convenience sample during the study period (November 2010 to March 2013), all adults presenting to the ED who were screened for possible sepsis when our research team was present were candidates for the study. Traditional Systemic Inflammatory Response Syndrome (SIRS) criteria were used for screening, including alterations in 1) temperature (> 38.0°C), 2) heart rate (> 90 beats/min), 3) respiratory rate (> 20 breaths/min), and 4) white blood cell count (>12*10^9^/L or <4*10^9^/L). Patients met initial screening criteria for the study if they met two of the four SIRS criteria and a nurse or physician provider identified a suspicion for infection. A “Code Sepsis” was triggered for patients with a systolic blood pressure of < 90 mm Hg, a mean arterial pressure of < 65 mm Hg, or a serum lactate of > 4 mmol/L, despite having received 30 mL/kg of intravenous fluids. Patients were studied if they met initial screening SIRS criteria or a “Code Sepsis” was triggered, and they had a serum lactate ordered.

Patients with bilateral arm or hand injuries were excluded, as were pregnant patients and prisoners. Patients were either initially approached for consent, or delayed consent (as approved by our IRB) was obtained following stabilization for subjects unable to provide consent at the time of data collection with no immediate next of kin available. Data were purged from subjects studied with delayed consent for whom consent was subsequently declined, and not included in the analyses. Our goal was to obtain optical data as soon as possible after identification in order to obtain MOx information before resuscitation was complete or definitive therapy provided. However, measurements made during this study did not interfere with standard assessment and treatment provided to these patients.

### Study protocol

Optical spectra were collected from patients with suspected sepsis and from the healthy control subjects by placement of the probe over the first dorsal interosseous (FDI) muscles on the back of the hand. Measurements were made with the hand resting on the bed, close to the level of the heart. Healthy control subjects were studied for 3 min at rest, while breathing room air. In patients with suspected sepsis, optical spectra were collected every 2–5 s continuously for a period of 8–15 minutes, as soon as possible following identification. Clinical data and ultimate clinical outcome for the subjects with suspected sepsis were obtained from the medical record.

### Measurements

#### Optical measurements

**Fig 1:**
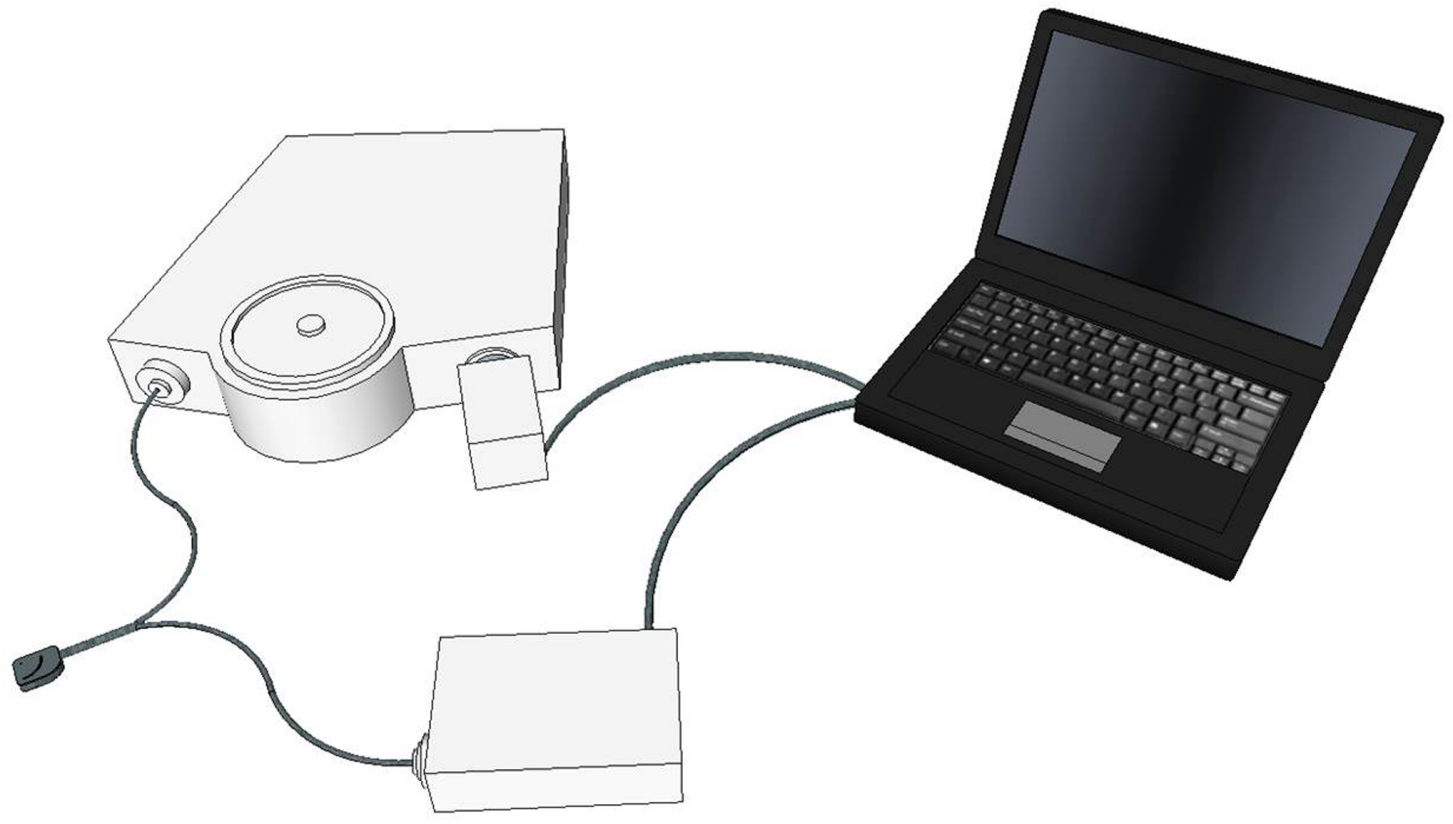
Schematic diagram of the optical spectral acquisition system configuration. A fiber-optic probe brings the incident light to the subject and returns reflected light to a spectrometer for computer processing.

**Fig 2:**
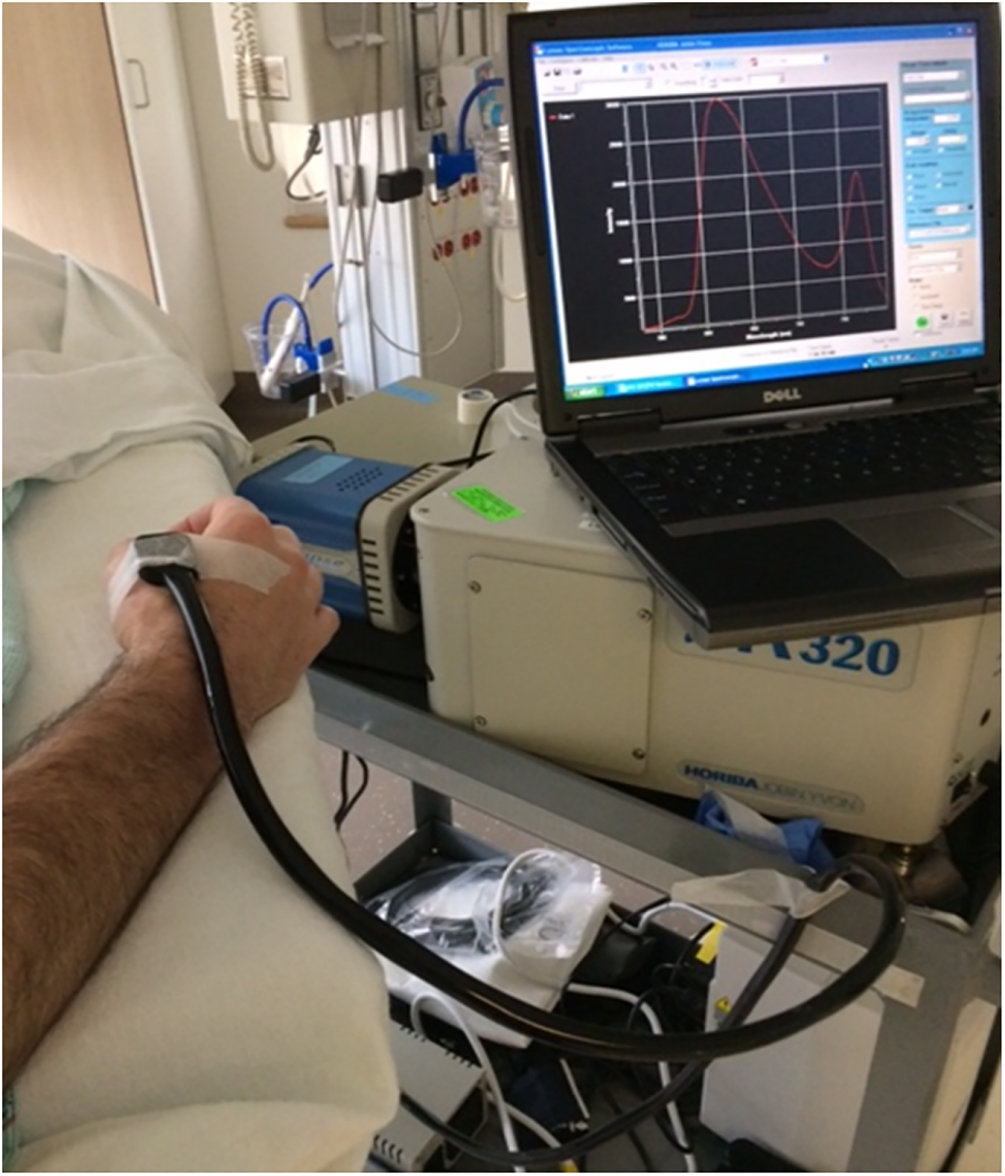
Photograph of the optical probe placed on a subject’s hand. The spectrometer and associated equipment is on a mobile cart at the bedside.

Optical spectra were collected with a custom-designed optical probe developed in our lab consisting of a set of illuminating optical fibers arranged in an arc, separated from a central set of detecting fibers arranged in a spot (Fiberoptic Systems, Inc., Simi Valley, CA). The distance between the illuminating and detecting optical fibers was set at 9, 11 or 13 mm in different optical probes. We have previously investigated sampling depth and separation of source and detecting optical fibers in tissue phantoms [16]. The choice of optical probe was based on physical characteristics of the subject and the quality of optical spectra obtained on initial testing, to obtain spectra with adequate signal-to-noise ratios from the deepest sampling depth possible. Spectra were obtained using an imaging spectrometer (iHR320, Horiba Jobin Yvon, Edison, NJ) coupled with a 256 x 1024-pixel thermoelectrically cooled CCD detector (Synapse, Horiba Jobin Yvon, Edison, NJ). A custom-designed LED-based light source (Innovations in Optics, Inc., Woburn, MA) provided illuminating light in the visible and NIR wavelength regions (500–800 nm). Fig 1 shows a schematic diagram of the optical system, and Fig 2 is a photograph illustrating the probe placement on a subject.

#### Determination of muscle oxygenation (MOx)

**Fig 3:**
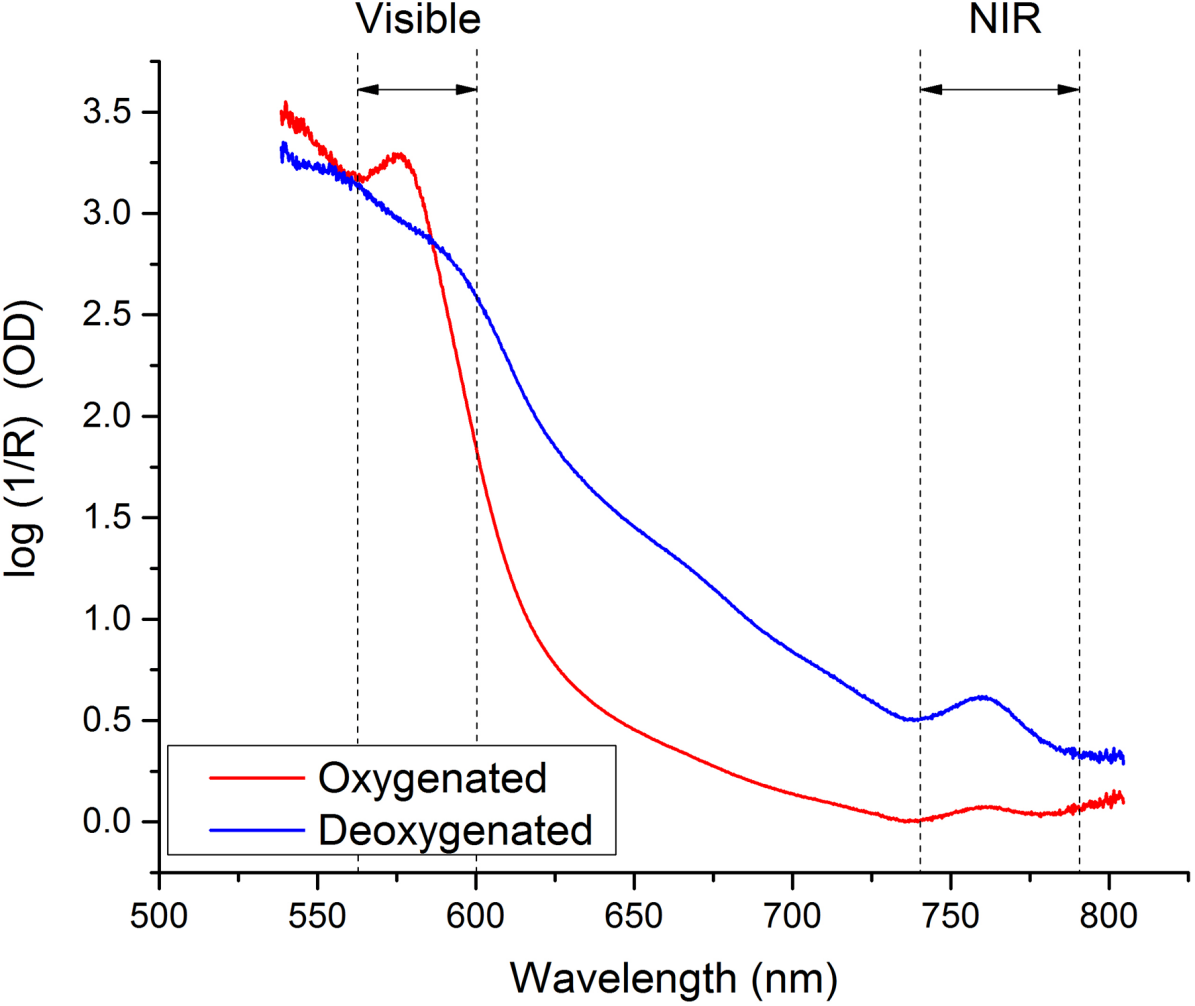
Reflectance spectra of oxygenated and deoxygenated first dorsal interosseous muscles (back of the hand) from a healthy adult. Only the highlighted regions in the visible and near infrared (NIR) are used in the analyses to determine muscle oxygenation.

MOx was determined from optical spectra using a multiwavelength analysis previously described [11] using MATLAB (R2014a, The MathWorks, Inc., Natick, MA) and PLS_Toolbox (v7.8, Eigenvector Research, Inc., Wenatchee, WA). Briefly, optical spectra were analyzed using an algorithm that was trained on a reference set of spectra previously obtained from the FDI muscles of 33 healthy subjects, separate from the control subjects in this study. Training set subjects had a wide range of body mass index (BMI) and skin tone so that particular skin tones or BMIs of the studied subjects had minimal effect on MOx measurements. Spectra acquired under fully oxygenated and fully deoxygenated conditions (using arm ischemia) in a training set subject are shown in Fig 3.

A MOx value was obtained from each spectrum acquired from sepsis patients and the study control subjects. Mean MOx values were calculated for each patient or control subject. Mean MOx was used to represent each subject in statistical analyses,

The Q-residuals test was used to determine whether or not MOx measurements made from spectra acquired from all subjects in this study were valid. The Q-residual test is an accepted chemometric test that determines if there is sufficient similarity in character between the given spectra and those in a training set that were used to build an analytical model [17]. Patients with Q-residuals higher than the 95% confidence interval of the Q-residuals in the training set were excluded from the study because their MOx readings were not valid.

#### Septic shock severity scale

Clinical data were collected from the medical record, including serum blood gases, hematocrits, lactates, and vital signs during the one hour period after the start of the optical measurements. Patient hospital length of stay, duration of intensive care unit admission, use of mechanical ventilatory support and mortality during that hospitalization were also recorded.

Determination of clinical shock severity was based on maximum heart rate, minimum systolic pressure, and maximum lactate values during the hour subsequent to the start of optical data collection, as previously described [18]. Table 1 shows the scoring system used to determine shock severity. Subjects were considered to have mild shock for scores up to 2, moderate for scores of 3–5, and severe if 6 or greater. This shock severity scale is intended to stratify patients at the time of the optical measurements based on vital signs and lactate, the most common indices currently used to diagnose and assess shock. We did not use a traditional scoring system such as the SOFA (Sequential Organ Failure Assessment) score [19], which includes the worst values collected over a 24 hour time period, as we specifically wanted to compare with clinical severity at the time of our MOx measurement. Traditional shock severity scores require additional information not available at all points in time during the day, such as creatinine and hematocrit.

**Table 1:** Clinical shock severity stratification scoring system.

	0 pts	1 pt	2 pts	3 pts	4 pts	5 pts
Maximum Heart Rate (beats/min)	< 100	100–120	> 120			
Minimum Systolic Pressure (mm Hg)	≥ 100	90–99	80–89	< 80		
Maximum Lactate (mmol/L)	< 1	1–1.9	2–3.9	4–7.9	8–11.9	≥ 12
Total score (pts) = Heart Rate + Systolic Pressure + Lactate						
Shock severity: Mild = 0–2 pts, Moderate = 3–5 pts; Severe = 6–10 pts						

Shock severity for each patient was not known during the optical spectral acquisition; it was determined post hoc with values taken from the medical record. Research personnel who retrieved data from the medical record to determine shock severity were blinded to MOx. In addition, research personnel who acquired optical spectra were not made aware of the parameters used in the shock severity scoring system.

#### Statistical analysis

Results are summarized as mean ± standard deviation (SD). Student’s *t*-tests were performed using Origin (v. 9.1.0, OriginLab Corp., Northampton, MA) for groups with more than 15 subjects [20]. The assumption of unequal variances was used [21].

## Results

**Fig 4:**
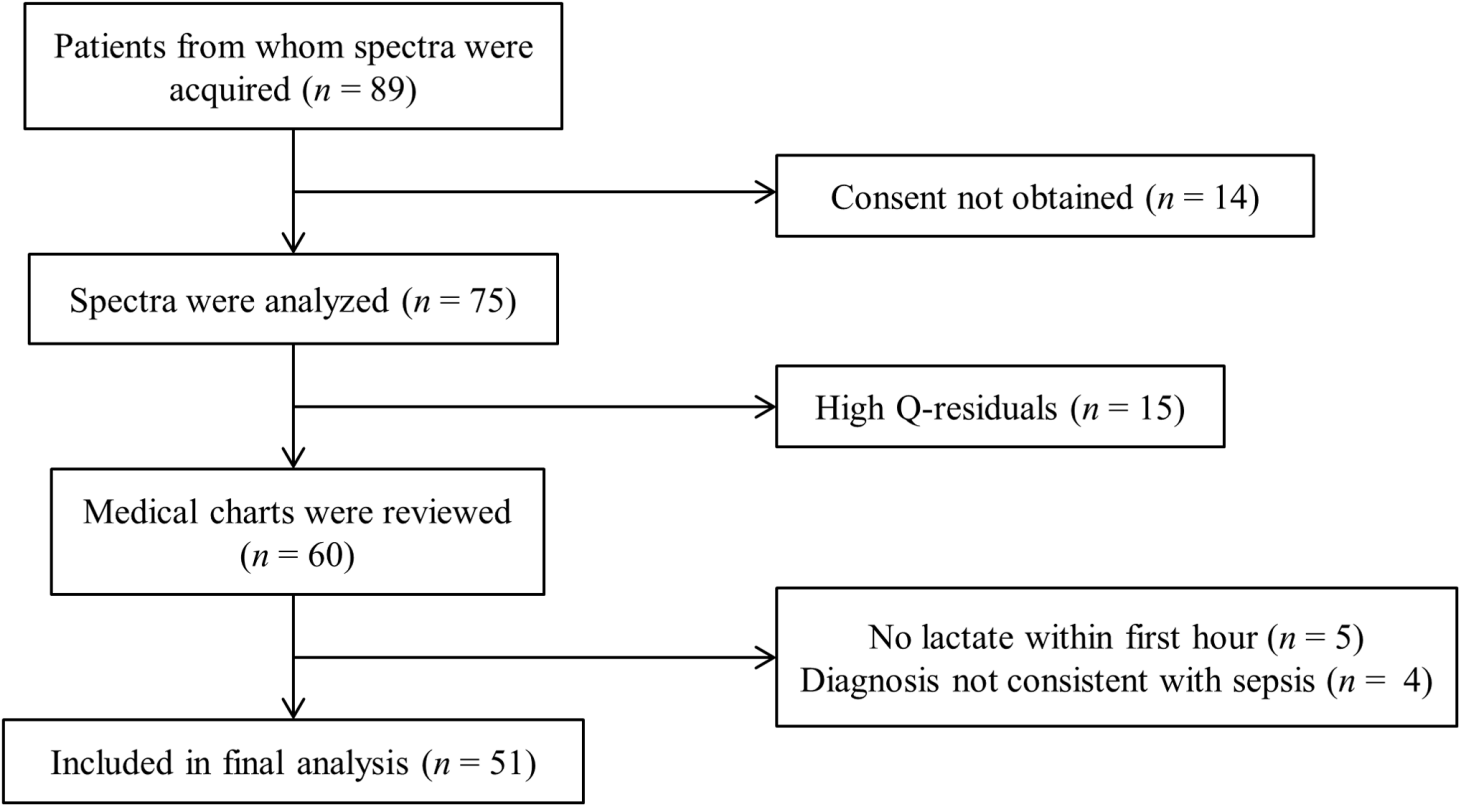
Patient enrollment. Muscle oxygenation (MOx) was measured from spectra acquired from patients. Q-residuals measure the similarity between patient spectra and the spectra that were used to train the algorithm that measures MOx. High Q-residuals (> 95% confidence interval) indicate a level of dissimilarity that renders MOx measurements invalid. MOx from 51 subjects was included in the final data set analyzed.

Optical spectra were acquired from 89 patients upon admission to the ED. The reasons for exclusion from the final data analysis are indicated in Fig 4. Consent was not obtained from 14 subjects either because they died before consent could be sought, no legal next of kin could be identified in a timely manner or were not available for consent, or consent was refused. Of the remaining 75 subjects, five were subsequently determined not to have a lactate drawn within the required time period, four were ultimately determined not to have evidence of sepsis (e.g., had diagnoses of cardiogenic shock or diabetic ketoacidosis), and 15 had Q-residuals above the acceptable cutoff (higher than the 95% confidence interval of the Q-residuals in the training set). Fifty-one subjects remained for analysis, as shown in Fig 4.

The control group consisted of 17 healthy adults. There were eight men and nine women with a mean age of 41.1 ± 9.5 years. As determined from subject questionnaires, none of the control subjects had evidence of cardiovascular disease; exercise-limiting pulmonary disease; diabetes mellitus; significant, uncontrolled hypertension; excessive ethanol intake; history of deep vein thrombosis or pulmonary embolism; cancer; rheumatoid disease; diagnosed peripheral arterial or vascular disease; or intermittent claudication.

Table 2 shows demographics of the three shock groups. Most patients had pneumonia (*n* = 18, 35.3%), soft tissue infections (*n* = 11, 21.6%), sepsis/ bacteremia without identified source (*n* = 10, 19.6%) or urinary tract infection (n = 5, 9.8%). Of those with sepsis/bacteremia, three had identified bacteremia, two isolates were classified as gram negative rods and one was a methicillin-sensitive Staphylococcus aureus. Other diagnoses included colitis (n = 2, 3.9%), pancreatitis (n = 2, 3.9%) and one each with septic arthritis, osteomyelitis and brain abscess (2.0%). Subjects ranged in age from 18 to 82 with a mean age of 52.5 ± 14.7 years. There were 17 female and 34 male subjects. Four patients had severe shock (8%), 30 had moderate shock (59%) and 17 had mild shock (33%).

**Table 2:** Subject demographics.

	Mild (*n* = 17)	Moderate (*n* = 30)	Severe (*n* = 4)
Sex			
Male (%)	11 (64.7)	19 (63.3)	4 (100)
Female (%)	6 (35.3)	11 (36.7)	0 (0)
Age (yr)	55.9 ±7.9	50.2 ± 19.7	56.0 ± 9.4
Primary Diagnoses			
Pneumonia (%)	8 (47.1)	9 (30.0)	1 (25.0)
Soft tissue infection (%)	5 (29.4)	6 (20.0)	0 (0)
Sepsis/bacteremia without source (%)	3 (17.6)	6 (20.0)	1 (25.0)
UTI (%)	0 (0)	5 (16.7)	0 (0)
Other (%)**	1 (5.9)	4 (13.3)	2 (50.0)
Initial Disposition			
ICU (%)	8 (47.1)	21 (70.0)	4 (100)
Floor (%)	9 (52.9)	9 (30.0)	0 (0)
Died (%)	1 (5.9)	6 (20.0)	1 (25.0)

UTI, urinary tract infection; ICU, intensive care unit.

**Other includes colitis (n = 2), pancreatitis (n = 2), septic arthritis, osteomyelitis and brain abscess.

**Fig 5:**
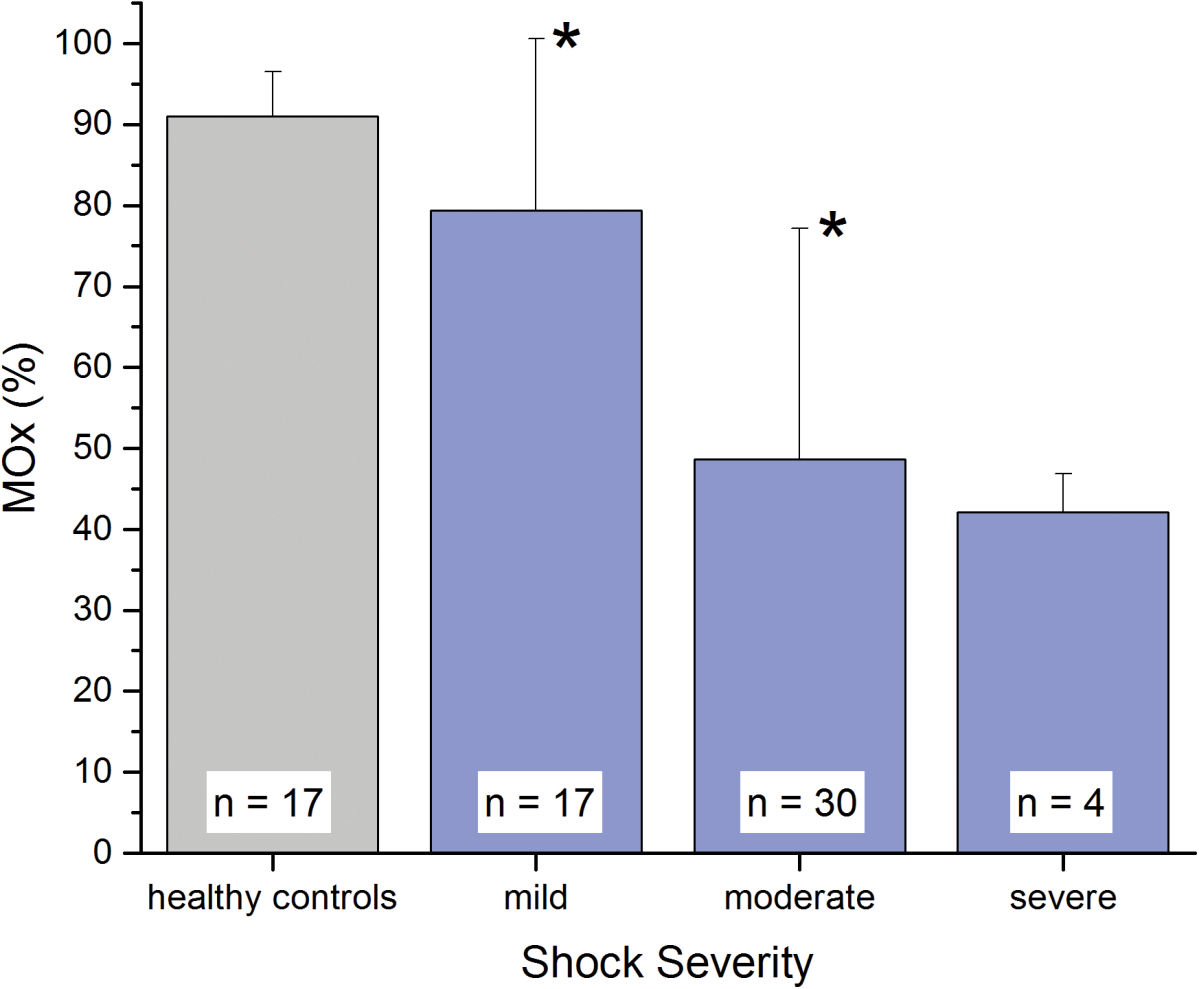
Muscle oxygenation (MOx) (mean ± SD) as a function of shock severity based on clinical scoring. Patients in mild and moderate shock categories were statistically significantly lower than healthy controls (*), and the mean MOx decrease between mild and moderate groups was also significant, p < 0.05. Statistical hypothesis testing was not performed on the severe group due to the small number of patients in that group.

Mean (± SD) MOx in the healthy control group was 91.0 ± 5.5% (*n* = 17). MOx for the control subjects ranged from 88.1% to 98.1% and MOx for the ill subjects ranged from 13.1% to 98.4%. MOx was associated with clinical shock severity, as shown in Fig 5 and Table 3. Patients with severe shock had a mean MOx of 42.2 ± 4.7%. MOx for patients with moderate shock was 48.6 ± 28.6%, and for mild shock MOx was 79.4 ± 21.2%. Two-sample *t-*tests revealed that there is a significant difference in mean MOx between control and mild, control and moderate, and mild and moderate groups (*p* < 0.05).

**Table 3:** Physiologic parameters of shock groups.

	Mild (*n* = 17)		Moderate (*n* = 30)		Severe† (*n* = 4)	
	mean ± stdev	median (25^th^, 75^th^ quartiles)	mean ± stdev	median (25^th^, 75^th^ quartiles)	mean ± stdev	median (25^th^, 75^th^ quartiles)
Hospital length of stay (days)	7.5 ± 9.4	3.0 (2.0, 9.0)	13.6 ± 19.1	7.5 (4.0, 12.0)	19.5 ± 28.8	6.0 (2.8, 22.8)
ICU length of stay (days)	2.0 ± 3.0	0 (0, 4.0)	3.2 ± 3.9	2 (0, 3.8)	18.0 ± 30.7	3.0 (2.8, 18.2)
Time on mechanical ventilation (days)	1.1 ± 1.9	0 (0, 2.0)	2.0 ± 3.5	0 (0, 2.0)	18.0 ± 30.7	3.0 (2.8, 18.3)
Maximum HR (bpm)	88.1 ± 15.1	88 (78, 92)	107 ± 22.9*	111 (94, 122)	111.2 ± 35.8	111 (77, 128)
Minimum SBP (mm Hg)	116.7 ± 18.3	111 (104, 133)	104 ± 16.2*	101 (92, 115)	83.0 ± 17.0	85 (66, 93)
Lactate (mM)	1.6 ± 0.7	2.0 (1.6, 2.9)	3.5 ± 2.7*	2.0 (1.7, 3.9)	10.2 ± 6.1	5.0 (2.8, 7.2)
Shock Index	0.77 ± 0.16	1.0 (0.62, 0.87)	1.04 ± 0.21*	1.0 (0.91, 1.21)	1.43 ± 0.75	1.0 (0.93, 1.52)
Minimum SpO_2_ (%)	96.4 ± 2.8	97 (95, 98)	97.5 ± 2.7	98 (98, 100)	96.0 ± 4.9	97 (91, 100)
MOx (%)	79.4 ± 21.2	90.2 (69.9, 96.1)	48.6 ± 28.6*	39.9 (24.8, 69.3)	42.2 ± 4.7	43.1 (41.2, 44.0)

*Means significantly different from mild group, *p* < 0.05.

stdev, standard deviation; ICU, intensive care unit; HR, heart rate; SBP, systolic blood pressure; SpO_2_, arterial saturation by pulse oximetry; MOx, muscle oxygenation.

†Statistical analysis not performed on severe group due to small number of subjects.

Hospital length of stay, ICU length of stay and time on mechanical ventilator support all trended higher with shock severity, but did not reach significance (Table 3). As expected, since heart rate and systolic blood pressure were two of the variables used to determine shock severity, heart rate increased with shock severity and systolic blood pressure decreased with shock severity. Correspondingly, shock index (defined as heart rate/systolic blood pressure) also significantly increased with severity of shock. Lactate values were significantly different between mild and moderate shock levels. The mean lactate was 1.6 ± 0.7 mmol/L, 3.5 ± 2.7 mmol/L, and 10.2 ± 6.1 mmol/L in the mild, moderate, and severe groups, respectively.

Overall, MOx trended higher in survivors compared with those who died (60.1 ± 30.6% vs. 51.3 ± 21.5%), but the difference did not reach statistical significance. MOx at ED admission was not different for those later admitted to the ICU (57.1 ± 31.3%) compared to those who were never admitted to the ICU during the hospital stay (59.1 ± 28.3%). Similarly, MOx at ED admission did not correlate to being placed on mechanical ventilation or not (57.5 ± 28.9% vs. 59.1 ± 29.7%, respectively). Table 3 also shows median values for these variables as well as the 25^th^ and 75^th^ quartiles.

## Discussion

The motivation for the current study was to evaluate the potential of a new noninvasive MOx measurement developed in our laboratory to identify patients with severe sepsis or septic shock. Patients with suspected sepsis were studied upon admission to the ED of a major academic regional referral and Level I trauma center. We found that MOx could distinguish patients in mild, and moderate shock, defined by degrees of tachycardia, hypotension, and lactate levels (Table 1). Results in this pilot study indicate that noninvasive MOx measurement has the potential to identify septic shock in its early stages.

MOx is associated with shock severity in a range of patients (Fig 5). The large error bars in Fig 5 are likely due to the stratification of patients into shock severity groups, rather than large errors in MOx measurement. We have previously estimated that there is about a 5% error in the MOx measurement [11]. Within a shock severity group, it is likely that clinical condition and stage of sepsis varied widely.

The ability of MOx to not only detect shock, but to stratify it, has important implications for emergency medicine and critical care. Upon admission to the ED or ICU, noninvasive MOx measurement may provide clinicians with a rapid indication of a patient’s cardiovascular and perfusion status. Patients in the mild shock severity group are of particular interest. The means of the mild shock group and the control group were significantly different (*p* < 0.05). Patients classified as mild had only slightly abnormal vital signs and/or slightly elevated lactate levels. Depressed MOx in this group indicates that MOx is a sensitive measure of tissue oxygenation with the potential to identify mild septic shock patients who may otherwise be missed.

In contrast to MOx, arterial saturation by pulse oximetry was not sensitive to shock in any of the severity groups (Table 3). Even in patients with severe septic shock, pulse oximetry values were in the normal range. While pulse oximeter values indicate the general health and status of the cardiopulmonary system, MOx is a direct indicator of tissue perfusion. Hemoglobin with arterioles, capillaries, and venules and myoglobin within muscle cells all contribute to the MOx measurement. MOx is a measure of the balance between oxygen supply and demand within muscle cells.

MOx measured at a single point in time did not correlate with mortality, ICU admission, or the need for mechanical ventilation during the hospital stay. This is not surprising because at the time of MOx measurement (early in the course of ED stay), patients had a wide range of condition and were in various stages of treatment. A larger, longitudinal study will be needed to evaluate MOx’s value in predicting patient outcomes.

Optical spectroscopy is attractive because it has the potential to provide noninvasive, continuous monitoring with a relatively inexpensive and small device that is easy to use. Peripheral muscle oxygenation may be the ideal tissue bed to assess adequacy of perfusion in illnesses that disrupt the cardiovascular system. When the cardiovascular system is impaired, blood flow is preferentially shunted to the most important organs in the body (brain, heart, liver, and kidneys) at the expense of peripheral muscle and skin. Low muscle oxygenation may serve as an early warning sign of inadequate systemic oxygenation.

Clinically available NIRS devices for tissue oxygenation (including cerebral oximeters) all measure only 2–6 selected NIR wavelengths of light. Since 2010, a currently available NIRS device (Inspectra 650) that measures tissue saturation has been used in several studies on patients with sepsis and septic shock [22]. The sensitivity of that device is questionable; tissue saturation (StO_2_) measured in healthy controls and patients with septic shock differed by only 3% [23], 2% [24], or 7% [25]. Thus there remains a need for a device that yields reliable and unambiguous tissue oxygenation values upon which clinicians can base clinical decisions [26–29].

Our approach differs significantly from currently available NIRS devices in that we include full-spectrum optical information from the visible and NIR spectral regions. Measurement in both spectral regions allows quantification of the relative concentrations of oxy- and deoxy- hemoglobin and myoglobin, resulting in accurate tissue oxygenation values in MOx [11]. Accurate and absolute MOx measurements will aid clinicians in recognizing tissue hypoxia definitively in different patients and in monitoring their responses to treatment.

The main limitations of this pilot study relate to the prototype system used for these measurements. Our MOx measurement is made using research equipment that is not yet clinically friendly and requires the constant presence of a member of our research team. Thus we used a convenience sample of study subjects and were only able to make single measurements of MOx in patients at early time points in their hospital admissions. We were limited in our ability to study all patients with suspected sepsis and to follow patient courses over time.

The small number of subjects enrolled did not allow an evaluation of the correlation between a single measurement of MOx and hospital course or outcome. Our current equipment was assembled for a research project at a single site, and is not yet designed for a larger multi-center study. In addition, our probe is not yet optimized for clinical data collection, resulting in 15 patients with poor spectral quality. Eleven of these patients were in the mild or moderate shock categories, leading to the conclusion that poor spectral quality was a result of operator error and not a systematic bias toward or against a particular shock category. Development of a user-friendly optical probe is underway.

The stage of illness for each patient at the time of MOx measurement was also not known and likely varied widely among patients in the study. Future studies will follow patients longitudinally, allowing evaluation of the value of MOx in assessment of a patient’s response to therapy.

## Conclusions

We demonstrate that a rapid, noninvasive measurement of muscle oxygenation is associated with the clinical assessment of shock severity in patients with suspected severe sepsis or septic shock. Notably, MOx in the mild shock group was statistically significantly lower than MOx in healthy controls. MOx holds promise in aiding early identification of shock in septic patients and in recognizing patients in mild shock that may otherwise be missed. Future studies designed to follow MOx throughout treatment for sepsis and septic shock may demonstrate utility in using MOx as a guide to initial resuscitation and subsequent management of these patients.
